# Effect of the Supine Position and Anesthesia on Nasal Anthropometric Measurements in Rhinoplasty Patients

**DOI:** 10.3390/diagnostics16010033

**Published:** 2025-12-22

**Authors:** Mustafa Said Tekin, Ekrem Karaca, Zeynep Bilge Yılmaz Dere, Ali Osman Korkmaz, Yusuf Muhammed Durna, Bahtiyar Hamit, Erkan Soylu

**Affiliations:** 1 Department of Otorhinolaryngology and Head & Neck Surgery, Medipol University Hospital, Istanbul Medipol University, Istanbul 34214, Turkey; 2 Department of Anatomy, School of Medicine, Istanbul Medipol University, Istanbul 34810, Turkey; 3 Clinical Anatomy PhD Program, Graduate School of Health Sciences, Istanbul Medipol University, Istanbul 34810, Turkey; 4 Vocational School, Istanbul Medipol University, Istanbul 34810, Turkey

**Keywords:** rhinoplasty, nasal anthropometry, supine position, anesthesia, photogrammetry, facial profile

## Abstract

**Background:** Body position and anesthesia may alter nasal anthropometric measurements, potentially affecting both preoperative assessment and intraoperative decisions in rhinoplasty. Quantifying these effects is essential for the accurate evaluation of nasal morphology. **Methods:** Thirty-five patients (28 females, 7 males; mean age 27.9 ± 6.95 years) undergoing primary rhinoplasty were prospectively included. Standardized lateral photographs were obtained in three conditions: upright, preanesthetic supine, and postanesthetic supine. Linear distances were measured using a digital caliper in a position replicating the photographic setup, and angular parameters were calculated from the photographs using ImageJ software (version 1.53, National Institutes of Health, Bethesda, MD, USA). Statistical analyses were performed to compare nasal measurements among the three positions. **Results:** The nasion–pronasale distance increased in the preanesthetic supine compared with the upright position (*p* < 0.001), while the pronasale–alar curvature distance remained unchanged (*p* = 0.984). The nasofrontal angle was higher upright (*p* < 0.001), whereas the nasolabial (*p* < 0.001) and nasomental (*p* = 0.010) angles were greater in the preanesthetic supine position. After anesthesia, both nasion–pronasale (*p* < 0.001) and pronasale–alar curvature (*p* = 0.001) distances increased further, while the nasofrontal angle remained higher upright (*p* < 0.001). **Conclusions:** Supine positioning and anesthesia significantly modify nasal profile parameters. Awareness of these predictable morphologic changes can help surgeons avoid misinterpretation and improve the accuracy of preoperative planning, intraoperative evaluation, and postoperative assessment.

## 1. Introduction

Rhinoplasty is one of the most frequently performed aesthetic surgical procedures worldwide [1]. Despite the accumulation of experience and continuous technical advancements, it remains a challenging operation [2,3]. The nose is located at the very center of the face and represents the most important structure that defines facial characteristics. Any modification of the nose therefore has a substantial impact on the overall facial appearance [4,5].

The nose, positioned centrally on the face, comprises a framework of bone and cartilage, lined internally by mucosa and enveloped externally by skin, subcutaneous tissue, and muscles of facial expression. This complex anatomy means that the nose is not a rigid structure, but one whose appearance shifts with facial movement [6]. Studies have shown, for example, that the length of the nose often increases during smiling, influenced by contraction of muscles such as the depressor septi nasi, which can also shorten the upper lip [7]. Moreover, anatomical investigations have revealed that fibers of depressor septi nasi and orbicularis oris may cross and attach to the medial crura of the alar cartilage, thereby contributing to dynamic pulling forces that affect nasal tip position during expression [8]. Such findings emphasize that nasal morphology is modulated not only by static structural elements, but also by soft tissue and muscle dynamics all critical for aesthetic evaluation and surgical planning.

An important factor that must be considered in rhinoplasty planning is the effect of body posture on the nasal profile. Surgeons often observe that the nasal contour assessed in the upright position during outpatient evaluation differs from that seen in the supine position on the operating table. However, it remains unclear whether this discrepancy is merely an optical illusion or results from positional changes in skin and soft tissue laxity [9,10]. This uncertainty underscores the necessity of objective anthropometric investigations comparing nasal morphology across different positions and conditions.

The present study was designed to objectively evaluate the impact of posture and anesthesia on nasal anthropometric parameters. Specifically, we compared linear and angular nasal measurements obtained in the upright position, the supine position before anesthesia induction, and the supine position after anesthesia induction. By documenting these variations within the same patient cohort, this study aimed to clarify whether nasal morphology remains consistent across different clinical settings and to provide surgeons with practical insights that may improve preoperative assessment and intraoperative decision-making in rhinoplasty.

## 2. Materials and Methods

### 2.1. Study Design and Participants

This prospective observational study was conducted at the Otorhinolaryngology Department of a tertiary referral hospital. A total of 35 patients (28 females, 7 males; age range: 18–50 years) scheduled for primary rhinoplasty due to nasal deformity were enrolled. Patients with a history of previous nasal surgery, congenital craniofacial anomalies, or acute nasal trauma were excluded. Written informed consent was obtained from all participants.

In addition to the routine rhinoplasty evaluation, each patient underwent a standardized facial and nasal assessment that included inspection of dynamic facial movements, baseline nasal symmetry, soft-tissue characteristics, and tip support mechanics to ensure consistent anatomical documentation prior to measurement procedures. Patients were recruited consecutively during the study period so that all eligible individuals meeting the inclusion criteria were invited without introducing selection bias. Because no robust preliminary data were available in the literature to support an a priori power calculation for posture- and anesthesia-related anthropometric changes, the final sample size reflected the number of eligible patients who presented during the study period. All assessments were performed in a controlled clinical environment to minimize external variability related to lighting, temperature, and patient positioning.

### 2.2. Photographic Protocol

Each patient was photographed in three standardized lateral profile positions:
Upright position in the outpatient clinic,
Supine position before anesthesia induction in the operating room,
Supine position after anesthesia induction in the operating room.

All photographs were obtained using the same digital camera (Nikon Coolpix, Nikon Corp., Tokyo, Japan) set at an approximate focal length of 50 mm (35 mm equivalent) and an aperture of around f/4.0, at a fixed distance of approximately 150 cm, against a plain dark background. The camera was positioned parallel to the sagittal plane at the level of the patient’s nasal profile. Lighting conditions were standardized with fluorescent illumination to minimize shadows.

To ensure strict reproducibility, a custom fixed-height tripod was used for all image acquisitions, and the camera height, distance, and angle were checked before every session. Patients were instructed to maintain a relaxed facial expression, keep their lips gently closed, and avoid smiling or raising the brows, as these actions may alter nasal tip position through the activation of perinasal muscles. The Frankfort horizontal plane was aligned parallel to the ground during upright imaging, while in the supine setting, the head was positioned on a neutral-support headrest to avoid unintended flexion or extension.

In the anesthetized group, photographs were taken 5 min after intravenous administration of rocuronium (0.6 mg/kg), following tracheal intubation and hemodynamic stabilization. All photographs were taken by the same investigator to ensure consistency.

### 2.3. Anthropometric Measurements

All linear measurements were performed directly on patients by a single investigator using a digital vernier caliper (BD500–200; BlueTec, Yongin, Republic of Korea) to ensure consistency. Inter- and intra-observer reliability analyses were not performed because all measurements were obtained by a single investigator. Upright measurements were obtained during clinical examination in the outpatient clinic. Supine measurements were obtained in two stages: pre-anesthetic measurements were taken immediately after patients were positioned on the operating table, whereas in the anesthetized group, measurements were performed 5 min after anesthesia induction, following tracheal intubation and hemodynamic stabilization. To minimize measurement variability, all caliper readings were obtained with the patient’s head maintained in a neutral position, and the instrument was applied perpendicular to the skin surface without compressing soft tissues.

Angular measurements were calculated from standardized lateral photographs taken in the same three positions using ImageJ software (version 1.53 National Institutes of Health, Bethesda, MD, USA). Before angular analysis, each photograph was calibrated to ensure identical pixel-to-millimeter scaling, and the same zoom level and viewing angle were used for all images. Anatomical points were selected manually using the software’s point-selection tool, and each landmark was identified according to predefined criteria to ensure reproducibility.

**Figure 1:**
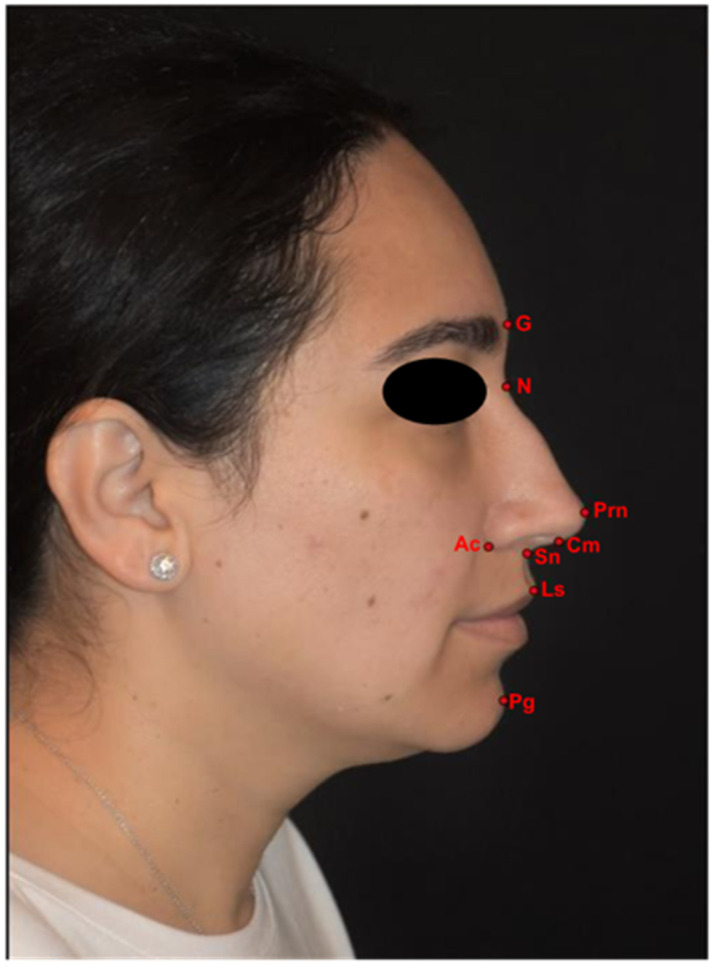
The anatomical landmarks utilised in the present study. G, glabella; N, nasion; Prn, pronasale; Cm, columella; Sn, subnasale; Ls, labiale superius; Pg, pogonion; Ac, alar curvature point.

The anatomical landmarks were defined according to the Farkas system [11] as follows (Figure 1):

Glabella (G): the most convex point of the forehead on the midface.
Nasion (N): the point in the midline of both the nasal root and the nasofrontal suture.
Pronasale (Prn): the most prominent point on the nasal tip.
Columella (Cm): the most anterior and inferior point on the apex of the nose.
Subnasale (Sn): the midpoint of the columella base.
Labiale superius (Ls): the midpoint of the vermilion line of the upper lip.
Pogonion (Pg): the most protrusive anterior sagittal midline point of the chin.
Alar curvature (Ac): the most lateral point in the curved baseline of each ala.

The calculated parameters included:

Linear measurements: nasion–pronasale distance, pronasale–alar curvature distance.

**Figure 2:**
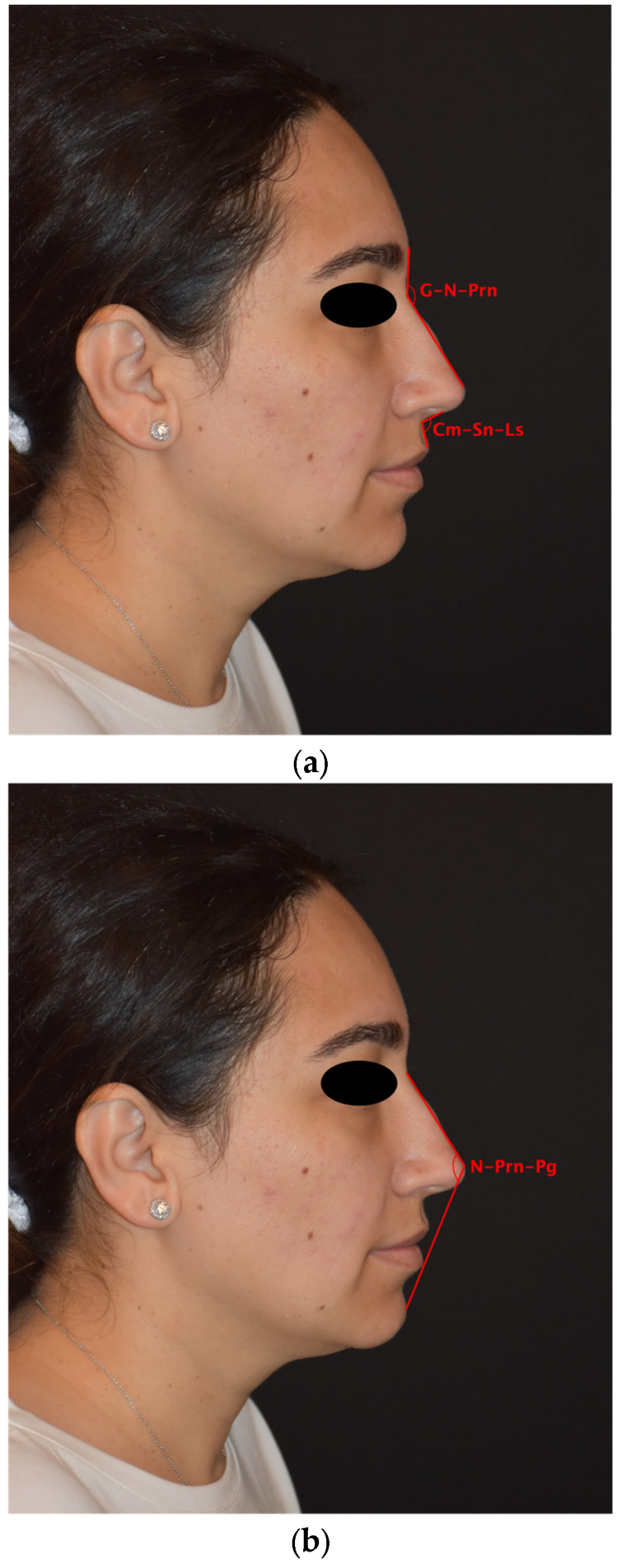
Angular measurements used in the analysis. (**a**) Nasofrontal angle (G–N–Prn: angle between glabella–nasion and nasion–pronasale lines) and nasolabial angle (Cm–Sn–Ls: angle between columella–subnasale and subnasale–labiale superius lines). (**b**) Nasomental angle (N–Prn–Pg: angle between nasion–pronasale and pronasale–pogonion lines). Abbreviations: G, glabella; N, nasion; Prn, pronasale; Cm, columella; Sn, subnasale; Ls, labiale superius; Pg, pogonion.

Angular measurements: nasofrontal angle (G–N–Prn: angle between glabella–nasion and nasion–pronasale lines), nasolabial angle (Cm–Sn–Ls: angle between columella–subnasale and subnasale–labiale superius lines), and nasomental angle (N–Prn–Pg: angle between nasion–pronasale and pronasale–pogonion lines) [12] (Figure 2a,b).

Due to the presence of the endotracheal tube, which displaces the upper lip and obscures the chin contour, nasolabial and nasomental angles could not be reliably assessed in the post-anesthetic supine position. Therefore, these parameters were evaluated only in the upright and pre-anesthetic supine positions.

### 2.4. Statistical Analysis

All statistical analyses were performed using Jamovi software (version 2.7.2.0 The Jamovi Project, Sydney, Australia). The normality assumption for continuous variables was assessed using the Shapiro–Wilk test. A paired sample *t*-test was performed to compare: (1) upright vs. pre-anesthetic supine position measurements, (2) pre- vs. post-anesthetic supine position measurements in supine position, and (3) upright vs. post-anesthetic supine position measurements, following confirmation of data normality. For descriptive statistics, the mean, median, standard deviation, and standard error values were calculated. In all analyses, a *p*-value less than 0.05 was considered statistically significant.

## 3. Results

A total of 35 patients (28 females and 7 males) were included in the study, with a mean age of 27.9 ± 6.95 years. When comparing the upright and preanesthetic supine positions, the nasion–pronasale distance was significantly greater in the preanesthetic supine position (*p* < 0.001), whereas no significant difference was observed in the pronasale–alar curvature distance (*p* = 0.984). Regarding angular measurements, the upright position demonstrated a significantly greater nasofrontal angle (*p* < 0.001), while the preanesthetic supine position showed significantly greater nasolabial (*p* < 0.001) and nasomental (*p* = 0.010) angles (Table 1).

In the comparison between preanesthetic and postanesthetic supine positions, both the nasion–pronasale (*p* = 0.029) and pronasale–alar curvature distances (*p* < 0.001) were significantly greater after anesthesia, while the nasofrontal angle showed no significant difference between the two supine measurements (*p* = 0.747) (Table 2).

When comparing the upright position with the postanesthetic supine position, both the nasion–pronasale (*p* < 0.001) and pronasale–alar curvature distances (*p* = 0.001) were significantly greater in the postanesthetic supine position. In contrast, the nasofrontal angle was significantly greater in the upright position compared to the postanesthetic supine position (*p* < 0.001) (Table 3).

## 4. Discussion

This study demonstrated that nasal anthropometric measurements obtained in the upright position differed significantly from those obtained in the supine position, particularly after anesthesia induction. Linear measurements such as the nasion–pronasale distance and angular parameters including the nasofrontal, nasolabial, and nasomental angles showed position-dependent variations, highlighting that nasal morphology is not constant across clinical settings. These findings emphasize that the nose evaluated preoperatively in the outpatient clinic may not represent the same anatomical structure encountered intraoperatively under general anesthesia.

A comprehensive preoperative assessment in rhinoplasty requires not only a detailed otorhinolaryngological examination but also standardized photographic documentation under appropriate lighting and distance, ensuring accurate facial and nasal analysis. In daily life, the upright position represents the natural posture of patients, and consequently, preoperative photographs are routinely obtained in this position. However, during surgery, the surgeon evaluates and manipulates the nose in the supine position, under general anesthesia, when all facial muscles are relaxed due to neuromuscular blockade. This discrepancy between the natural upright evaluation and the intraoperative supine-anesthetized condition is of critical importance, as it directly influences the surgeon’s intraoperative perception of nasal dimensions and angles.

Our findings demonstrate that nasal measurements obtained in the upright position differed significantly from those in the supine position, particularly after anesthesia induction. In practical terms, the nose analyzed preoperatively in the clinic is not identical to the one observed intraoperatively under anesthesia; both angular and linear parameters undergo measurable changes. This discrepancy carries critical implications for surgical planning. Previous studies have shown that even small variations in the nasolabial angle can influence nasal tip rotation and the overall perception of nasal aesthetics [13,14]. Considering that millimetric differences in nasal morphology may lead to substantial aesthetic consequences, recognition of these positional and anesthetic changes is essential to ensure accurate and predictable surgical outcomes.

The mechanisms underlying these alterations are not fully established, but based on our observations, we hypothesize that several factors may contribute. Gravity likely exerts different forces depending on body posture, acting vertically from superior to inferior in the upright position but anteroposteriorly in the supine position, thereby influencing nasal length and angles. In addition, we believe that general anesthesia with neuromuscular blockade induces complete relaxation of facial muscles, reducing soft tissue tension and subsequently modifying the nasal contour. Together, these factors may at least partly explain why the intraoperative nasal appearance in anesthetized supine patients differs from the preoperative upright evaluation.

Kim et al. evaluated nasal profiles in the erect position using lateral cephalograms and in the supine position using paranasal computed tomography, reporting that the nasofrontal angle was significantly wider in the supine position, whereas no differences were detected in the nasolabial and nasomental angles [15]. Angular parameters such as the nasofrontal, nasolabial, and nasomental angles are widely recognized as critical determinants of nasal and facial aesthetics. The nasofrontal angle plays a pivotal role in radix assessment and influences the perception of dorsal contour and nasal length [16,17]. The nasolabial angle is a key indicator of tip rotation, where even minor variations can influence the perceived nasal tip position [18,19], whereas the nasomental angle contributes to the harmony between the nose and chin, thereby affecting the overall balance of the facial profile [20]. In our study, angular measurements were analyzed on standardized profile photographs, while linear nasal distances were obtained directly on patients during clinical examination, and, importantly, both sets of measurements were assessed before and after anesthesia in the supine position. This design enabled objective documentation of not only positional changes but also anesthesia-related soft tissue alterations, offering clinically relevant insights for surgical planning and aesthetic evaluation.

This study has some limitations that should be acknowledged. First, it was conducted in a single center with a relatively small sample size, which may limit the generalizability of the findings. Second, due to the presence of the endotracheal tube, the nasolabial and nasomental angles could not be reliably assessed in the post-anesthetic supine position, restricting the evaluation of these parameters. Additionally, the generalizability of the results may be limited by the demographic characteristics of the study cohort, which consisted predominantly of young female patients.

From a clinical perspective, our findings highlight the importance of recognizing posture- and anesthesia-related changes in nasal morphology during rhinoplasty. Surgeons should be aware that the intraoperative nasal appearance under anesthesia does not fully represent the preoperative upright evaluation, and therefore preoperative photographs obtained in the natural upright position should be available in the operating room as a reference. Preoperative upright photographs represent the most accurate depiction of the nasal form, and postoperative photographs should also be obtained in the same standardized upright position to ensure reliable comparison. This practice may help achieve more accurate, harmonious, and patient-satisfying outcomes. In standard operating room settings, maintaining a neutral head position with gentle and consistent occipital support may help reduce positional variability during supine assessment. Future studies with larger cohorts, diverse ethnic populations, and three-dimensional imaging techniques are warranted to further validate and expand upon these observations. Another line of research could be to determine whether measurement errors, such as those described, truly matter. The natural healing process can alter the outcome more than measurement errors, even under conditions of impeccable surgical technique.

## 5. Conclusions

This study demonstrated that some nasal anthropometric measurements differ significantly between upright and supine positions, with additional alterations observed after anesthesia induction. These findings indicate that both posture and anesthesia influence nasal morphology, underscoring the importance of incorporating upright preoperative photographs into intraoperative decision-making. In practical terms, the nose evaluated preoperatively in the clinic is not identical to the one encountered during surgery under anesthesia. Surgeons should therefore keep upright preoperative photographs available in the operating room and refer to them when necessary, in order to ensure that intraoperative maneuvers lead to results that are realistic and harmonious with the patient’s overall facial appearance.

## Figures and Tables

**Table 1:** Comparison of upright and preanesthetic supine position measurements.

Measurement	Value		*p*-Value
	Upright Position	Preanesthetic Supine Position	
Nasion-to-pronasale distance	48.7 ± 4.50	49.2 ± 4.53	<0.001
Pronasale-to-alar curvature distance	32.6 ± 3.22	32.6 ± 3.00	0.984
**Measurement**	**Value**		***p*** **-Value**
	**Upright Position**	**Preanesthetic Supine Position**	
Nasofrontal angle	148.0 ± 8.52	145.1 ± 7.97	<0.001
Nasolabial angle	99.1 ± 12.53	102.3 ± 12.27	<0.001
Nasomental angle	128.1 ± 5.49	128.5 ± 5.40	0.010

**Table 2:** Comparison of supine position preanesthetic and postanesthetic measurements.

Measurement	Value		*p*-Value
	Preanesthetic Supine Position	Postanesthetic Supine Position	
Nasion-to-pronasale distance	49.2 ± 4.53	49.5 ± 4.64	0.029
Pronasale-to-alar curvature distance	32.6 ± 3.00	33.0 ± 3.24	<0.001
**Measurement**	**Value**		***p*** **-Value**
	**Preanesthetic Supine Position**	**Postanesthetic Supine Position**	
Nasofrontal angle	145.1 ± 7.97	145.0 ± 8.24	0.747

**Table 3:** Comparison of upright and postanesthetic supine position measurements.

Measurement	Value		*p*-Value
	Upright Position	Postanesthetic Supine Position	
Nasion-to-pronasale distance	48.7 ± 4.50	49.5 ± 4.64	<0.001
Pronasale-to-alar curvature distance	32.6 ± 3.22	33.0 ± 3.24	0.001
**Measurement**	**Value**		***p*** **-Value**
	**Upright Position**	**Postanesthetic Supine Position**	
Nasofrontal angle	148.0 ± 8.52	145.0 ± 8.24	<0.001

## Data Availability

The data presented in this study are available from the corresponding author upon reasonable request. Due to privacy and ethical restrictions, data are not publicly available.
